# A three ion channel genes-based signature predicts prognosis of primary glioblastoma patients and reveals a chemotherapy sensitive subtype

**DOI:** 10.18632/oncotarget.12462

**Published:** 2016-10-04

**Authors:** Hao-Yuan Wang, Ji-Ye Li, Xiu Liu, Xiao-Yan Yan, Wen Wang, Fan Wu, Ting-Yu Liang, Fan Yang, Hui-Min Hu, Heng-Xu Mao, Yan-Wei Liu, Shi-Zhong Zhang

**Affiliations:** ^1^ Department of Neurosurgery, Zhujiang Hospital, Southern Medical University, Guangzhou, China; ^2^ The National Key Clinical Specialty, The Engineering Technology Research Center of Education Ministry of China Guangdong Provincial, Key Laboratory on Brain Function Repair and Regeneration, Department of Neurosurgery, Zhujiang Hospital, Southern Medical University, Guangzhou, China; ^3^ Department of Neurosurgery, Beijing Tiantan Hospital, Capital Medical University, Beijing, China; ^4^ Beijing Neurosurgical Institute, Capital Medical University, Beijing, China; ^5^ Chinese Glioma Cooperative Group (CGCG), Beijing, China; ^6^ Department of Radiation Therapy, Beijing Tiantan Hospital, Capital Medical University, Beijing, China; ^7^ Center for Brain Disorders Research, Capital Medical University, Beijing, China

**Keywords:** alpha-fetoprotein, antigen epitope, heat shock protein 70, functional peptide, immunity

## Abstract

Increasing evidence suggests that ion channels not only regulate electric signaling in excitable cells but also play important roles in the development of brain tumor. However, the roles of ion channels in glioma remain controversial. In the present study, we systematically analyzed the expression patterns of ion channel genes in a cohort of Chinese patients with glioma using RNAseq expression profiling. First, a molecular signature comprising three ion channel genes (KCNN4, KCNB1 and KCNJ10) was identified using Univariate Cox regression and two-tailed student's t test conducted in overall survival (OS) and gene expression. We assigned a risk score based on three ion channel genes to each primary Glioblastoma multiforme (pGBM) patient. We demonstrated that pGBM patients who had a high risk of unfavorable outcome were sensitive to chemotherapy. Next, we screened the three ion genes-based signature in different molecular glioma subtypes. The signature showed a Mesenchymal subtype and wild-type IDH1 preference. Gene ontology (GO) analysis for the functional annotation of the signature showed that patients with high-risk scores tended to exhibit the increased expression of proteins associated with apoptosis, immune response, cell adhesion and motion and vasculature development. Gene Set Enrichment Analysis (GSEA) results showed that pathways associated with negative regulation of programmed cell death, cell proliferation and locomotory behavior were highly expressed in the high-risk group. These results suggest that ion channel gene expression could improve the subtype classification in gliomas at the molecular level. The findings in the present study have been validated in two independent cohorts.

## INTRODUCTION

Ion channels, membrane proteins expressed in all living cells, create pathways for charged ions, including calcium (Ca^2+^), potassium (K^+^), sodium (Na^+^), and chloride (Cl^−^) ions. During the last few years, ion channels have been demonstrated to play critical roles in gene expression, immune response, cell volume regulation, cell migration, and cell proliferation [1–3]. Particularly, there is increasing evidence that ion channels are involved in the progression of human cancers [4–7] and ion channel genes-based signature has the potential role in prognosis of breast cancer and lung cancer [8, 9].

Glioblastoma multiforme (GBM) is the most common primary central nervous system tumor with a current median survival of approximately 15 months [10]. Despite continuous progress in neurosurgery, the infiltrative behavior of gliomas precludes complete tumor resection and is certainly the primary reason for the poor clinical outcome for patients [11, 12]. Ion channel genes have been demonstrated that play an important role in brain tumor metastasis [13, 14].

In this study, we used RNASeq datasets from CGGA and a set of 280 ion channel genes to identify an ion genes-based signature for clinical outcomes of primary GBM (pGBM) patients. We then built a predictive model based on the three ion genes that correlated overall survival (OS) and validated the model by applying it to the TCGA and REMBRANDT datasets. The three ion genes signature identified patients who had a high risk of unfavorable outcome were sensitive to chemotherapy.

## RESULTS

### Identification of a three ion channel genes signature for prognosis in pGBM patients

**Figure 1:**
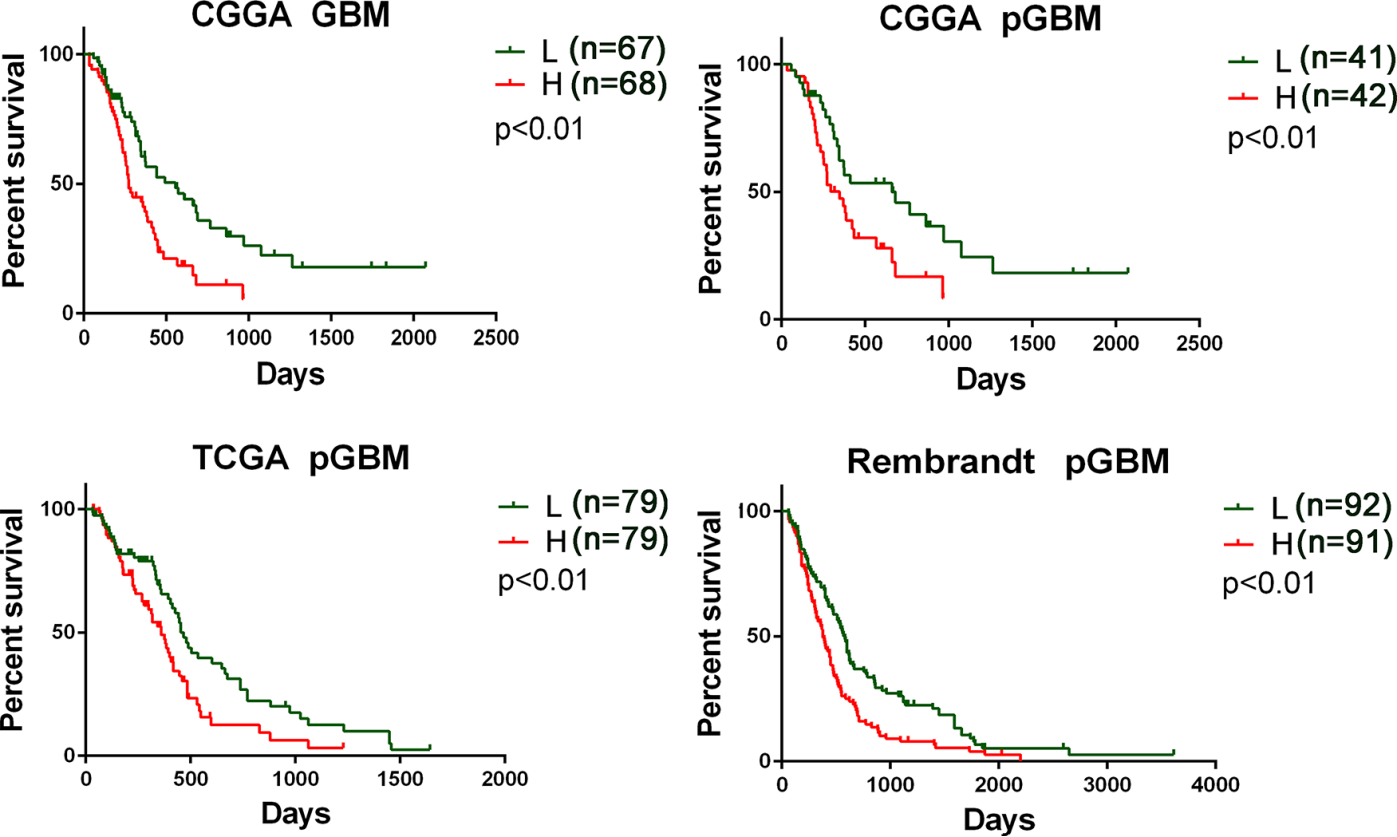
Prognostic values of three ion channel genes-based signature for patients in training and validation datasets Patients in low risk group showed a better prognosis than those in high risk group according to the signature risk score in the CGGA dataset (A–B), the TCGA data (C), and the Rembrandt data (D). L, low risk group; H, high risk group; pGBM, primary GBM.

**Figure 2:**
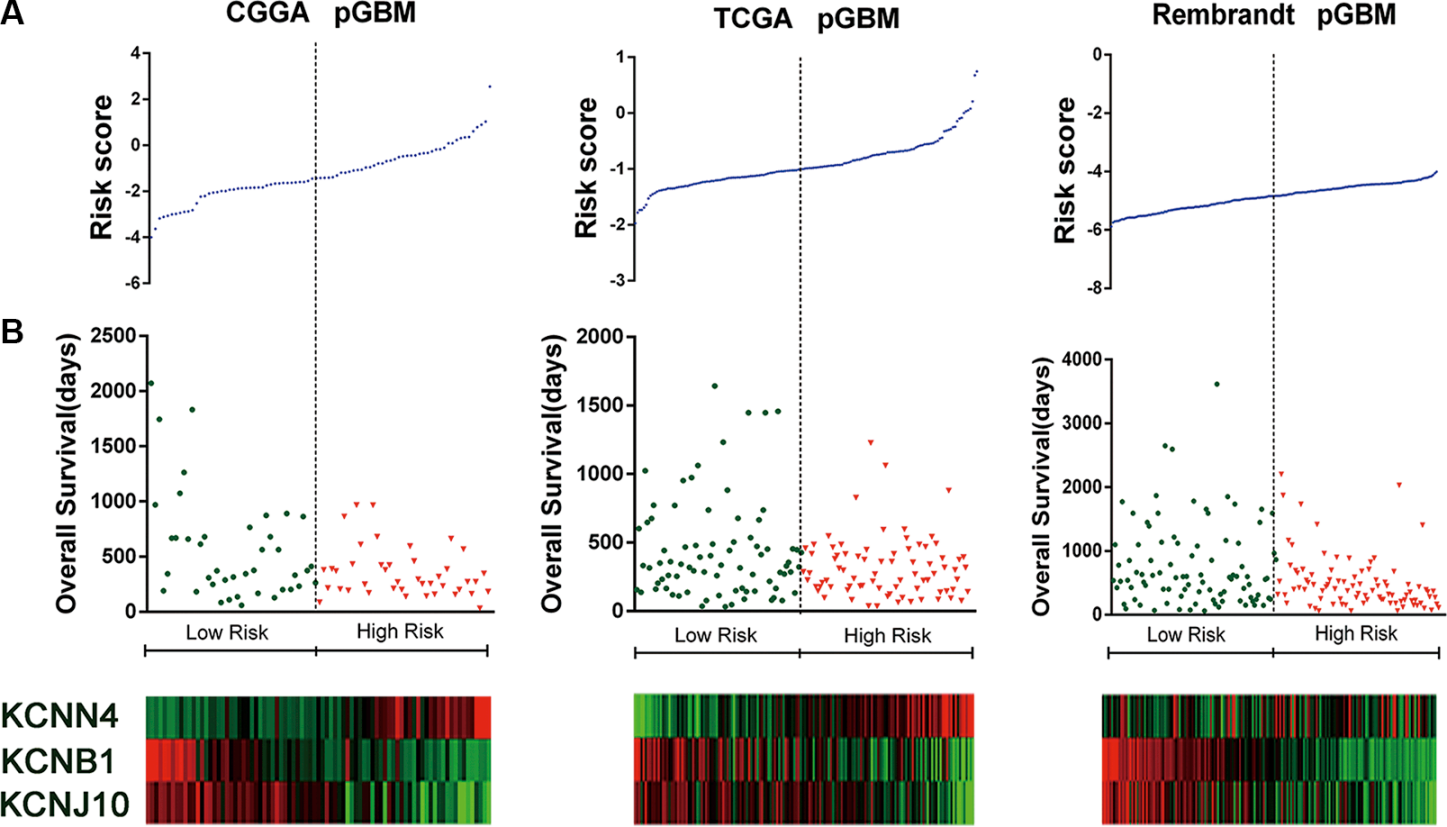
Distributions of risk score of pGBM and OS of their patients in the three datasets (**A**) Signature risk score distribution. (**B**) Patient overall survival duration. (C) Expression of the three ion channel genes-based signature along the risk score. Red indicates high expression and green indicate low expression.

A total of 280 ion channel genes were collected for the present study (Additional file 1: Supplementary Table S1). To identify ion channel genes which were associated with grade progression, we first compared genome expression in grade II or grade IV gliomas with that in grade III gliomas (II VS III and III VS IV) in CGGA dataset of gliomas, then a two-sided log-rank test was used to analyze each ion genes in GBM patients. Finally, three channel genes (KCNN4, KCNB1 and KCNJ10) were identified to be significantly correlated with malignant progression and associated with OS (Supplementary Figure S1, Supplementary Table S2). The expression value of KCNN4 is upregulated and KCNB1 and KCNJ10 are downregulated in gliomas. We then applied the three genes as a signature to develop a risk score formula by using the risk score method. The risk score for each patient was then calculated. Using the median risk score as the cutoff value, the patients were successfully divided into a high risk group and a low risk group. The patients with the high risk score had a shorter median OS than patients with the low risk score in GBM and pGBM. (*p* < 0.001) (Figure 1A–1B). The risk score and OS distribution were shown in Figure 2A and 2B.

We then determined the dependence of the signature of clinicopathological and molecular parameters in pGBM patients from CGGA database by multivariate Cox regression analyses. All the parameters (Table 1) were selected based on our clinical experiences that were related to prognosis. We found that the risk score, chemotherapy and radiotherapy status were statistically associated with OS. Multivariate Cox analysis indicated that the risk score was an independent prognostic factor (*p* < 0.05) (Table 2).

**Table 1:** Characteristics of patients in low risk and high risk group in three datasets

		CGGA		*p*	TCGA		*p*	Rembrandt		*p*
		LR	HR		LR	HR		LR	HR	
Sample size		41	42		79	79		92	91	
	F	15	15		26	32		24	24	
Gender	M	26	27	> .05	53	47	> .05	48	37	> .05
	NA	0	0		0	0		20	30	
Age		46.3 ± 11.8	54.0 ± 12.3	< .01	58.5 ± 14.4	62.8 ± 10.1	< .05	NA	NA	
	Y	31	27		74	78		NA	NA	
Radiotherapy	N	9	7	> .05	5	1	> .05	NA	NA	
	NA	2	8		0	0		NA	NA	
	Y	30	21		58	57		NA	NA	
Chemotherapy	N	10	12	> .05	20	22	> .05	NA	NA	
	NA	1	9		0	0		NA	NA	
	WT	32	41		71	78		NA	NA	
IDH1 mutation	Mut	9	1	< .05	7	1	> .05	NA	NA	
	NA	0	0		1	0		NA	NA	
	WT	37	38		3	1		NA	NA	
ATRX	Mut	4	4	> .05	73	75	> .05	NA	NA	
	NA	0	0		3	3		NA	NA	
KPS		NA	NA		75.5 ± 14.9	77.6 ± 14.9	> .05	NA	NA	

*P* value for age and KPS: *t* test; *p* value for others: chi-square test or Fisher's exact test; LR, low risk group; HR, high risk group; F, female; M male; NA, not available; WT, wild type; Mut, mutation; KPS, Karnofsky performance status; Y, underwent radiotherapy/chemotherapy; N, not underwent radiotherapy/chemotherapy.

**Table 2:** Factors associated with OS in the Cox regression analysis for pGBM patients from the CGGA dataset

	Univariate Cox Regression			Multivariate Cox Regression		
variable	HR	95% CI	*p* value	HR	95%CI	*p* value
Gender (Female vs. Male)	1.321	0.735–2.375	> 0.05			
Age (< 45 vs. > 45)	1.221	0.691–2.159	> 0.05			
Risk score (Low vs. High)	2.042	1.152–3.620	< 0.05	2.133	1.105–4.115	< 0.05
Chemotherapy (Yes vs. No)	0.359	0.196–0.656	< 0.01	0.434	0.222–0.848	< 0.05
Radiotherapy (Yes vs. No)	0.468	0.239–0.917	< 0.05	0.585	0.266–1.289	> 0.05
IDH1 status (MUT vs. WT)	0.308	0.097–0.974	> 0.05			
ATRX status (MUT vs. WT)	0.444	0.062–3.192	> 0.05			

WT, wild type; Mut, mutation. Yes, underwent radiotherapy/chemotherapy; No, not underwent radiotherapy/chemotherapy.

### Validation of the prognostic value of the three genes signature in two additional datasets

Further, we validated the independent predictive power of the three genes signature in the TCGA and REMBRANDT datasets. For the 158 and 183 pGBM in TCGA and REMBRANDT datasets, we used the same β value obtained from the training set to calculate the risk scores. Patients were also divided into high risk group and low risk group according to the risk score (cutoff: median risk score). The prognostic value of the signatures was validated by the two datasets (*p* < 0.01 for all the two datasets, Figure 1C–1D). The risk score and OS distribution were also shown in Figure 2A–2B.

We then validated the dependence of the signature of clinicopathological and molecular parameters in pGBM patients from TCGA datasets by multivariate Cox regression analyses. The parameters related to prognosis were selected (Table 1). We found that the risk score, age and IDH1 status were statistically associated with OS. Multivariate Cox analysis validated that the risk score was an independent prognostic factor (*p* < 0.05) (Supplementary Table S3).

### The three ion genes-based signature assisted predicting the efficacy of chemotherapy in pGBM patients

**Figure 3:**
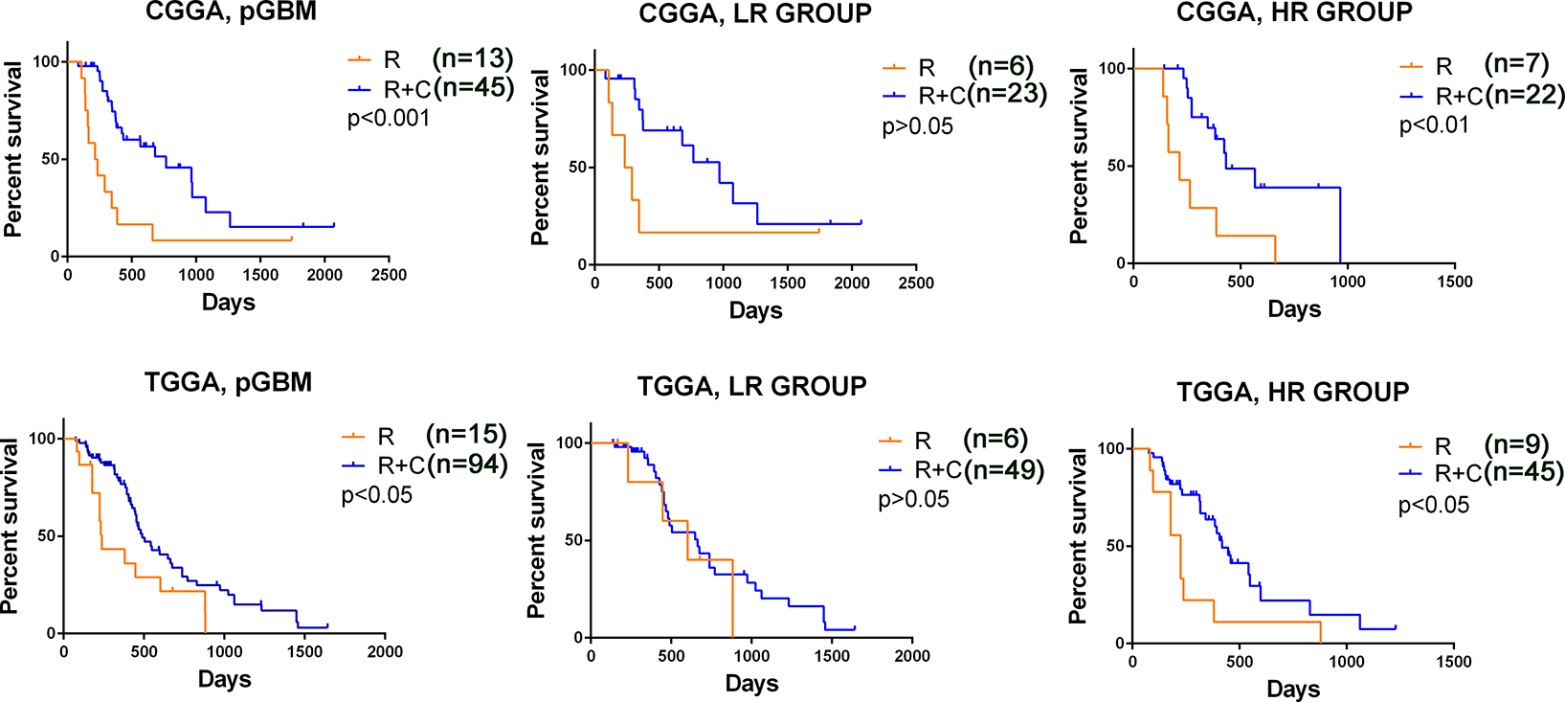
The signature predicted the efficacy of radiotherapy with or without chemotherapy in pGBM patients (A, D) pGBM patients in CGGA and TCGA treated with RT + CT showed a better prognosis than those with RT alone. (B, E) Benefit of RT + CT was observed in the high risk group with significantly improved OS (*p* < 0.05). (C, F) The addition of CT to RT did not improve OS of patients in the low risk group (*p* > 0.05). RT, radiotherapy; CT, chemotherapy; LR, low risk group; HR, high risk group.

To determine whether the three genes signature assists in predicting the efficacy of postoperative radiotherapy (RT) and chemotherapy (CT) in pGBM patients, we extracted the therapeutic information available for 83 pGBM patients in the CGGA datasets (Figure 3A, *p* < 0.001). According to risk scores, 27 patients (6 patients underwent RT and 21 patients underwent RT+CT) were stratified to the high-risk group and the other 31 patients (7 patients underwent RT and 24 patients underwent RT+CT) to the low-risk group. Among high-risk pGBM patients, a more favorable survival benefit was observed in the RT+CT treatment group compared to the RT alone group (Figure 3C, *p* < 0.01), while OS did not differ significantly between RT+CT and RT alone treatment group among low-risk pGBM patients (Figure 3B, *p* > 0.05).

We then used 158 pGBM patients treated with standard RT with or without CT in TCGA databases to confirm the therapeutic predictive value of the signature (Figure 3D, *p* < 0.05). Similarly, RT+CT was only beneficial for the high-risk pGBM patients (21 RT/57 RT+CT) but not for the low-risk pGBM patients (15 RT/59 RT+CT) (Figure 3E–3F). The findings indicate that high-risk pGBM patients were sensitive to chemotherapy.

### The three genes signature showed a subtype preference

**Figure 4:**
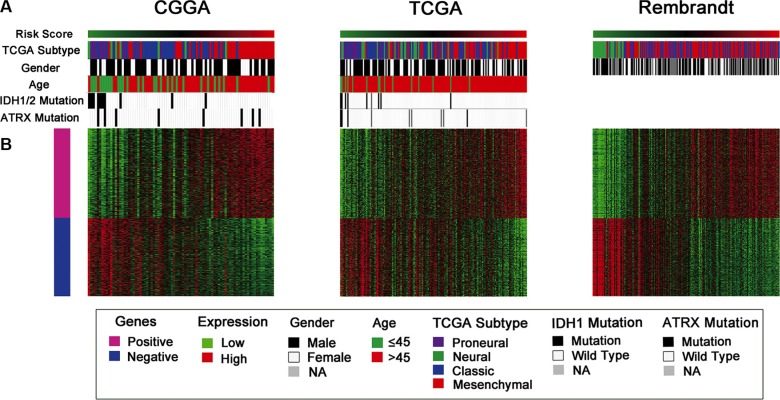
Distribution of molecular and clinical pathological features for pGBM patients in three datasets (**A**) The high risk score tumors displayed Mesenchymal subtype and wild-type IDH1 preference. (**B**) The differentially expressed genes were shown arranged from the low to high risk score. Pink represents the high expression of genes in the high risk group; blue represents the low expression of the genes in high risk group.

Considering the promising potential of the three ion genes signature in predicting clinical therapies, we next screened the expression of the three genes signature in different molecular glioma subtypes. We found that tumors of patients with high risk scores obviously displayed TCGA Mesenchymal subtype and wild-type IDH1 preference in the three datasets of CGGA, TCGA and REMBRANDT (*P* < 0.001, respectively) (Figure 4A–4B).

### Functional Annotation of the three genes signature

**Figure 5:**
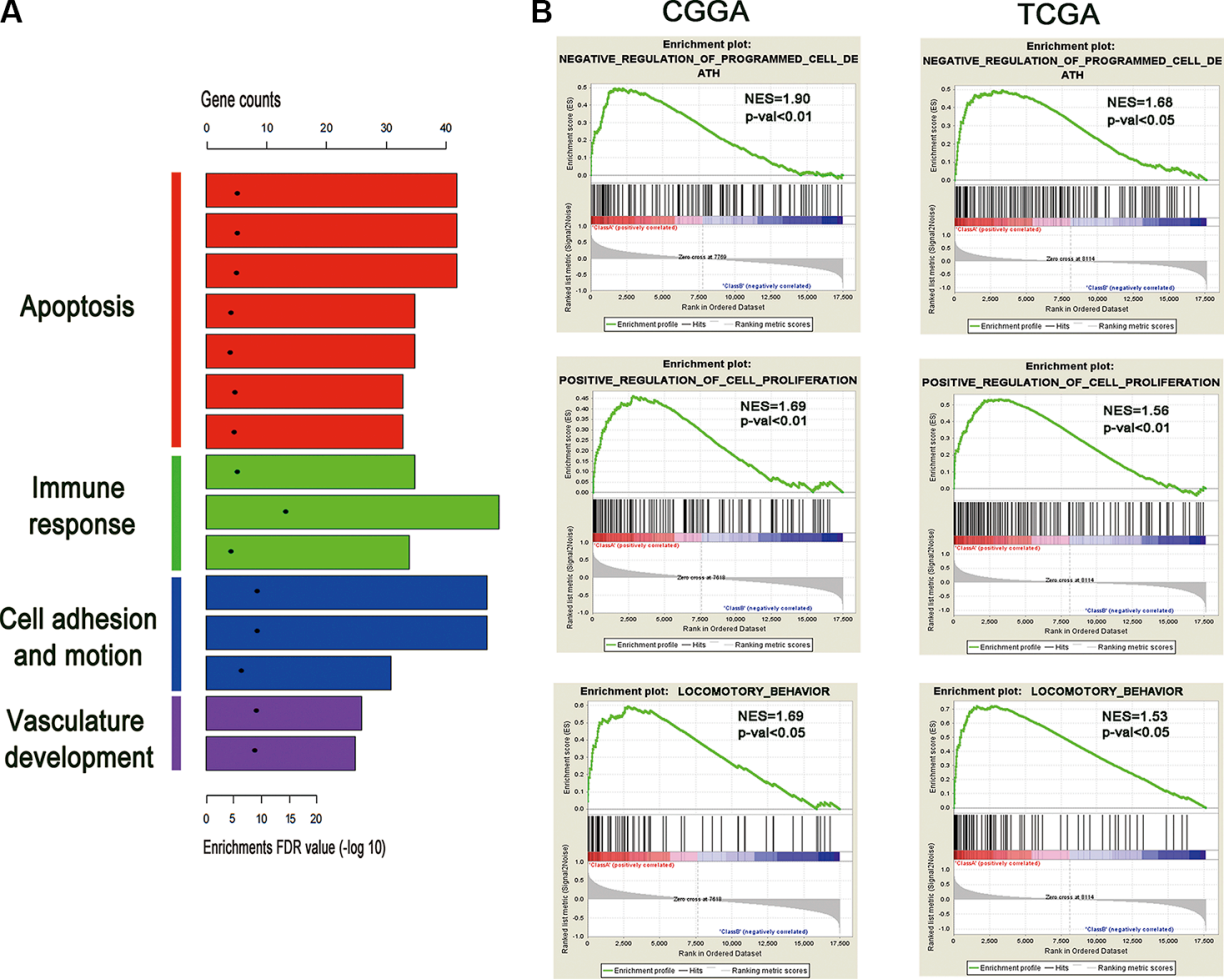
Functional annotation of the high risk group (**A**) GO analysis revealed the significant association of the genes with increased expression in high risk group with four main pathways. Column height: gene counts; point height: enrichment *p* value. (**B**) Three representative plots of GSEA from enriched pathways in high risk group, analyzed by gene set enrichment analysis of CGGA and TCGA RNAseq data.

In order to find out the functional basis of the notable difference in prognosis, we also performed SAM on high and low risk group in three datasets. After 1000 times of permutation test, those genes with false discovery rate (FDR) < 0.01 were considered to be differentially expressed between the two groups. By screening the top 1000 increased expression genes, the overlapped genes (424 genes with increased expression in high risk group, Additional file 1: Supplementary Table S4) were chosen for further analysis. Gene ontology (GO) analysis revealed that the associated genes, among those highly expressed in the high-risk group, were primarily associated with the apoptosis, immune response, cell adhesion and motion and vasculature development (Figure 5A). Furthermore, GSEA results showed that pathways associated with negative regulation of programmed cell death, cell proliferation and locomotory behavior were highly expressed in the high-risk group (Figure 5B).

## DISCUSSION

In recent years, the gene expression-based molecular classification of gliomas has rapidly developed [15, 16]. Previous studies suggested that a multiple ion channel genes-based risk signature can provide a more statistical analysis than a single gene [8, 9]. The aim of our study was to identify a small group of genes whose expression predicts survival in pGBM gliomas and can be readily measured. We first identified three ion channel genes (KCNN4, KCNB1 and KCNJ10) significantly associated with OS of pGBM patients, then we designated the three genes as the three ion gene-based signature. The ion gene signature was then demonstrated as a promising prognostic molecular signature for predicting the OS in three independent cohorts from different regions worldwide. We also observed the preferred expression of the ion gene signature in mesenchymal subtype and wild-type IDH1.

In recent years, it has become increasingly clear that downregulation of ion channels is an important mechanism in drug resistance via impairment of cell death [17, 18]. As drug resistance to chemotherapy is the major challenge in the treatment of pGBM, it will be increasingly important to identify patients who do not benefit from chemotherapy and who may be candidates for early treatment with chemotherapy. In our study, among high-risk pGBM patients, a more favorable survival benefit was observed in the RT+CT treatment group compared to the RT alone group (*p* < 0.01). However, OS did not differ significantly between RT+CT and RT alone treatment group among low-risk pGBM patients. The findings indicate that the low-risk patients should avoid unnecessary chemotherapy treatment since it usually causes toxic side-effects.

Several studies reported that ion channel genes play the important role of apoptosis, proliferation, cell migration [2, 14, 19–21]. In our studies, GO analysis revealed that patients with high-risk scores are likely associated with the apoptosis, immune response, cell motion and vasculature development. GSEA results showed that pathways associated with negative regulation of programmed cell death, cell proliferation and locomotory behavior were highly expressed in the high-risk group.

Previous studies have investigated the prognostic roles of the three ion channel genes in the process of tumors. These three genes are all included in the family of the potassium channels. KCNB1 (Kv2.1) is the principal voltage-gated potassium (Kv) channel underlying delayed-rectifier currents (IDR) in most mammalian brain neurons. We identified KCNB1 was associated with malignant progression and outcome in gliomas. Moreover, KCNB1 plays an important role in the autophagy induction via activation of the ERK signaling pathway (manuscript in submission).

KCNN4 (KCa3.1) channels belong to the Ca^2+^-activated potassium channel superfamily and the activation of these channels is dependent on conformational changes in calcium calmodulin [22]. Previous researches have revealed that KCNN4 channels regulate cell cycle progression and cell growth in human endometrial cancer and prostate cancer cells [23, 24]. More importantly, KCa3.1 channels are involved in the infiltrative behavior of glioblastoma and significantly enhances glioma invasion [21, 25, 26].

KCNJ10 (Kir4.1), is the predominant K^+^ channel in mature astrocytes and responsible for establishing the astrocytes negative resting membrane potential [27]. KCNJ10 was demonstrated as overexpressed in glioma cells and promoted cell differentiation and inhibited growth in gliomas [28].

Limitation of this study includes the fact that it is a retrospective research and three ion genes-based signature was generated from the small population of the validation datasets. For clinical application, a larger independent dataset in a prospective study is required.

In summary, we identified a novel three ion channel genes-based signature with independent prognostic significance for pGBM that can be a useful tool for identifying patients who would most benefit from chemotherapy treatment.

## MATERIALS AND METHODS

### Patients and samples

All glioma samples included in our study were from the Chinese Glioma Genome Atlas (CGGA), which were composed of 109 grade II gliomas, 41 grade III gliomas and 83 primary GBM. The patients underwent surgical resection between January 2006 and December 2009. Patients were eligible for the study if the diagnosis of glioma was established histologically according to the 2007 WHO classification. These patients underwent surgery and were followed-up at Beijing Tiantan hospital. Clinicopathological data, including gender, age, pathologic diagnosis and the results of molecular analysis were obtained.

Whole transcriptome sequencing of 233 gliomas were obtained from Chinese Glioma Genome Atlas (CGGA) database (http://www.cgga.org.cn) [29]. The other two datasets were downloaded from the repository for the Cancer Genome Atlas (TCGA) dataset (http://cancergenome.nih.gov) and the molecular brain neoplasia data (REMBRANDT, http://caintegrator.nci.nih.gov/rembrandt).

The definition of human ion channel genes was obtained from GeneCards [30, 31] and IUPHAR-DB [32]. In total, we collected 280 ion channels, including voltage-dependent and non-voltage-dependent ion channels.

### Signature development

Patients surviving more than 90 days were eligible for the study since too short survival time were more likely resulted from severe complication rather than glioma occurrence. Overall survival (OS) was calculated as the interval from the day of first surgery to death or the end of follow-up. Firstly, an unpaired two-tailed Student's *t* test was used to compare the expression value of each gene in grade II or grade IV gliomas with that in grade III gliomas (II VS III and III VS IV) in three datasets of gliomas (CGGA, TCGA, and REMBRANDT). Then, the prognostic difference of a certain gene was calculated by the Univariate COX regression analysis with log-rank test by packages (survival) of R to get the corresponding Hazard Ratio (HR) in each grade. Then genes which were associated with grade progression and significant prognostic value (*p*-value < 0.05) were used to developed a linear combination of the gene expression level (expr) weighted by the regression coefficient derived from the univariate Cox regression analysis (β). As a result, we identified three ion channel genes, which were then used as a signature for prediction utility assessment. Based on the three-gene signature, the risk score for each patient was calculated as follows:

$${\text{Risk score}=\text{expr}}_{\text{gene}1}\times \beta_{\text{gene}1}+{\text{expr}}_{\text{gene}2}\times \beta_{\text{gene}2}+\ldots +{\text{expr}}_{\text{gene n}}\times \beta_{\text{gene n}}$$

According to this model, the patients having higher risk scores were expected to have poor OS. Patients of every grade in the training dataset were stratified into a high-risk or a low-risk group by using the 50th percentile risk score as the cut-off. We used the same β in the validation sets.

### DAVID analysis of associated genes in gliomas

Significant analysis of microarray (SAM) was performed in pGBM to identify differently expressed genes, followed GO [33] and GSEA analysis of the differently expressed genes highly expressed in the high risk score group.

### Statistical analysis

Statistical analysis was performed using Graphpad Prism 5.0 by Student's *t* test or Mann–Whitney test. The associations between risk score and clinicopathological features were tested by Pearson Chi-Square test. Kaplan–Meier and log-rank methods were used to compare OS curves using SPSS version 20. Statistically significant variables in the univariable analysis were included in multivariable analysis using Cox proportional hazards model. All statistical tests were two-sided. A difference was considered significant when *p* < 0.05.

## SUPPLEMENTARY MATERIALS FIGURES AND TABLES






