# Supplementary material for: A three ion channel genes-based signature predicts prognosis of primary glioblastoma patients and reveals a chemotherapy sensitive subtype
Source: Oncotarget. 2016 Oct 4;7(46):74895–903. doi: 10.18632/oncotarget.12462 (PMC5342710; doi:10.18632/oncotarget.12462)
Supplement: Supplementary file 1 [file oncotarget-07-74895-s001.pdf]

A three ion channel genes-based signature predicts prognosis of primary glioblastoma patients and reveals a chemotherapy sensitive subtype

Supplementary Materials

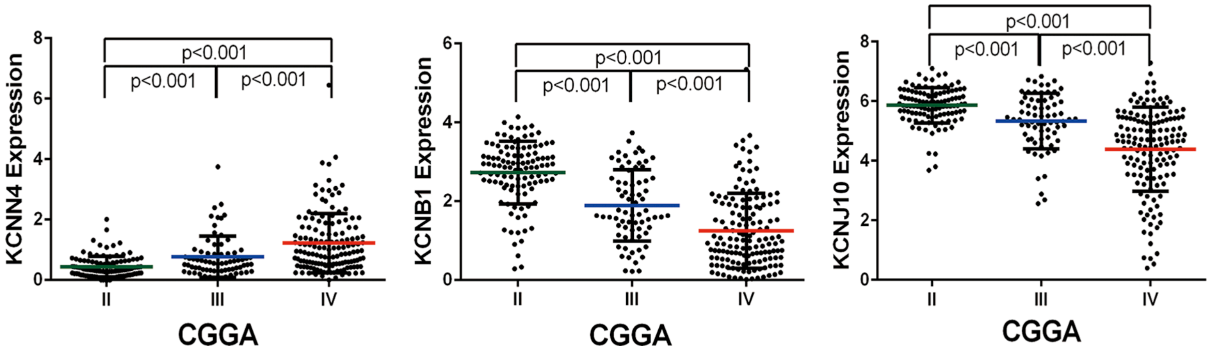

Supplementary Figure S1: Expression of the three ion channel genes in the CGGA datasets. Each spot represents the gene expression value of the individual patient. Line in the middle is the mean expression value. II, WHO grade II; III, WHO grade III; IV, WHO grade IV.

Supplementary Table S1: Ion channel genes involved in this study. See Supplementary_Table_S1

Supplementary Table S2: Three genes associated significantly with overall survival time in pGBM from the CGGA dataset

Symbol	Hazard radio	95% Confidence interval	*P*-value
KCNN4	1.459	(1.209, 1.760)	7.93E–05
KCNB1	0.619	(0.474, 0.809)	4.35E–04
KCNJ10	0.775	(0.671, 0.897)	5.94E–04

Supplementary Table S3: Factors associated with OS in the Cox regression analysis for pGBM patients from the TCGA dataset

	Univariate Cox Regression			Multivariate Cox Regression		
variable	HR	95% CI	*p* value	HR	95%CI	*p* value
Gender (Male vs. Female)	0.854	0.579–1.261	> 0.05			
Preoperative KPS score (≥ 80 vs.< 80)	0.830	0.510–1.350	>0.05			
Age at diagnosis (< 45 vs. ≥ 45)	2.446	1.130–5.292	< 0.05	1.697	0.701–4.107	>0.05
Risk score (Low vs. High)	1.703	1.145–2.531	<0.001	1.492	1.002–2.233	<0.05
IDH1 status (MUT vs. WT)	0.308	0.097–0.974	<0.05	0.518	0.140–1.914	> 0.05
ATRX status (MUT vs. WT)	0.444	0.062–3.192	> 0.05			
Chemotherapy (Yes vs. No)	1.182	0.768–1.819	> 0.05			
Radiotherapy (Yes vs. No)	2.358	0.745–7.459	> 0.05			

WT, wild type; Mut, mutation. Yes, underwent radiotherapy/chemotherapy; No, not underwent radiotherapy/chemotherapy.

Supplementary Table S4: 424 genes with increased expression in high risk group. See Supplementary_Table_S4
