# Supplementary material for: A three ion channel genes-based signature predicts prognosis of primary glioblastoma patients and reveals a chemotherapy sensitive subtype
Source: Oncotarget. 2016 Oct 4;7(46):74895–903. doi: 10.18632/oncotarget.12462 (PMC5342710; doi:10.18632/oncotarget.12462)
Supplement: Supplementary file 2 [file oncotarget-07-74895-s002.docx]

Table S1. Ion channel genes involved in this study

| *Gene symbol* | Gene title | Channel type |
| --- | --- | --- |
| *ANO1* | anoctamin 1, Calcium activated chloride channel | Calcium activated chloride channel |
| *ANO2* | anoctamin 2, Calcium activated chloride channel | Calcium activated chloride channel |
| *CACNA1A* | calcium channel, voltage-dependent, P/Q type, alpha 1A subunit | Voltage-gated calcium channels |
| *CACNA1B* | calcium channel, voltage-dependent, N type, alpha 1B subunit | Voltage-gated calcium channels |
| *CACNA1C* | calcium channel, voltage-dependent, L type, alpha 1C subunit | Voltage-gated calcium channels |
| *CACNA1D* | calcium channel, voltage-dependent, L type, alpha 1D subunit | Voltage-gated calcium channels |
| *CACNA1E* | calcium channel, voltage-dependent, R type, alpha 1E subunit | Voltage-gated calcium channels |
| *CACNA1F* | calcium channel, voltage-dependent, L type, alpha 1F subunit | Voltage-gated calcium channels |
| *CACNA1G* | calcium channel, voltage-dependent, T type, alpha 1G subunit | Voltage-gated calcium channels |
| *CACNA1H* | calcium channel, voltage-dependent, T type, alpha 1H subunit | Voltage-gated calcium channels |
| *CACNA1I* | calcium channel, voltage-dependent, T type, alpha 1I subunit | Voltage-gated calcium channels |
| *CACNA1S* | calcium channel, voltage-dependent, L type, alpha 1S subunit | Voltage-gated calcium channels |
| *CACNA2D1* | calcium channel, voltage-dependent, alpha 2/delta subunit 1 | Voltage-gated calcium channels |
| *CACNA2D2* | calcium channel, voltage-dependent, alpha 2/delta subunit 2 | Voltage-gated calcium channels |
| *CACNA2D3* | calcium channel, voltage-dependent, alpha 2/delta subunit 3 | Voltage-gated calcium channels |
| *CACNA2D4* | calcium channel, voltage-dependent, alpha 2/delta subunit 4 | Voltage-gated calcium channels |
| *CACNB1* | calcium channel, voltage-dependent, beta 1 subunit | Voltage-gated calcium channels |
| *CACNB2* | calcium channel, voltage-dependent, beta 2 subunit | Voltage-gated calcium channels |
| *CACNB3* | calcium channel, voltage-dependent, beta 3 subunit | Voltage-gated calcium channels |
| *CACNB4* | calcium channel, voltage-dependent, beta 4 subunit | Voltage-gated calcium channels |
| *CACNG1* | calcium channel, voltage-dependent, gamma subunit 1 | Voltage-gated calcium channels |
| *CACNG2* | calcium channel, voltage-dependent, gamma subunit 2 | Voltage-gated calcium channels |
| *CACNG3* | calcium channel, voltage-dependent, gamma subunit 3 | Voltage-gated calcium channels |
| *CACNG4* | calcium channel, voltage-dependent, gamma subunit 4 | Voltage-gated calcium channels |
| *CACNG5* | calcium channel, voltage-dependent, gamma subunit 5 | Voltage-gated calcium channels |
| *CACNG6* | calcium channel, voltage-dependent, gamma subunit 6 | Voltage-gated calcium channels |
| *CACNG7* | calcium channel, voltage-dependent, gamma subunit 7 | Voltage-gated calcium channels |
| *CACNG8* | calcium channel, voltage-dependent, gamma subunit 8 | Voltage-gated calcium channels |
| *CATSPER1* | cation channel, sperm associated 1 | CatSper and Two-Pore channels |
| *CATSPER2* | cation channel, sperm associated 2 | CatSper and Two-Pore channels |
| *CATSPER3* | cation channel, sperm associated 3 | CatSper and Two-Pore channels |
| *CATSPER4* | cation channel, sperm associated 4 | CatSper and Two-Pore channels |
| *CATSPERB* | catsper channel auxiliary subunit beta | CatSper and Two-Pore channels |
| *CATSPERD* | catsper channel auxiliary subunit delta | CatSper and Two-Pore channels |
| *CATSPERG* | catsper channel auxiliary subunit gamma | CatSper and Two-Pore channels |
| *CHRNA1* | cholinergic receptor, nicotinic, alpha 1 (muscle) | Nicotinic acetylcholine receptors |
| *CHRNA10* | cholinergic receptor, nicotinic, alpha 10 (neuronal) | Nicotinic acetylcholine receptors |
| *CHRNA2* | cholinergic receptor, nicotinic, alpha 2 (neuronal) | Nicotinic acetylcholine receptors |
| *CHRNA3* | cholinergic receptor, nicotinic, alpha 3 (neuronal) | Nicotinic acetylcholine receptors |
| *CHRNA4* | cholinergic receptor, nicotinic, alpha 4 (neuronal) | Nicotinic acetylcholine receptors |
| *CHRNA5* | cholinergic receptor, nicotinic, alpha 5 (neuronal) | Nicotinic acetylcholine receptors |
| *CHRNA6* | cholinergic receptor, nicotinic, alpha 6 (neuronal) | Nicotinic acetylcholine receptors |
| *CHRNA7* | cholinergic receptor, nicotinic, alpha 7 (neuronal) | Nicotinic acetylcholine receptors |
| *CHRNA9* | cholinergic receptor, nicotinic, alpha 9 (neuronal) | Nicotinic acetylcholine receptors |
| *CHRNB1* | cholinergic receptor, nicotinic, beta 1 (muscle) | Nicotinic acetylcholine receptors |
| *CHRNB2* | cholinergic receptor, nicotinic, beta 2 (neuronal) | Nicotinic acetylcholine receptors |
| *CHRNB3* | cholinergic receptor, nicotinic, beta 3 (neuronal) | Nicotinic acetylcholine receptors |
| *CHRNB4* | cholinergic receptor, nicotinic, beta 4 (neuronal) | Nicotinic acetylcholine receptors |
| *CHRND* | cholinergic receptor, nicotinic, delta (muscle) | Nicotinic acetylcholine receptors |
| *CHRNE* | cholinergic receptor, nicotinic, epsilon (muscle) | Nicotinic acetylcholine receptors |
| *CHRNG* | cholinergic receptor, nicotinic, gamma (muscle) | Nicotinic acetylcholine receptors |
| *CLCA1* | chloride channel accessory 1 | Calcium activated chloride channel |
| *CLCA2* | chloride channel accessory 2 | Calcium activated chloride channel |
| *CLCA3* | chloride channel accessory 3 | Calcium activated chloride channel |
| *CLCC1* | chloride channel CLIC-like 1 | Mid-1-related chloride channel |
| *CLCN1* | chloride channel, voltage-sensitive 1 | Voltage-sensitive chloride channel |
| *CLCN2* | chloride channel, voltage-sensitive 2 | Voltage-sensitive chloride channel |
| *CLCN3* | chloride channel, voltage-sensitive 3 | Voltage-sensitive chloride channel |
| *CLCN4* | chloride channel, voltage-sensitive 4 | Voltage-sensitive chloride channel |
| *CLCN5* | chloride channel, voltage-sensitive 5 | Voltage-sensitive chloride channel |
| *CLCN6* | chloride channel, voltage-sensitive 6 | Voltage-sensitive chloride channel |
| *CLCN7* | chloride channel, voltage-sensitive 7 | Voltage-sensitive chloride channel |
| *CLCNKA* | chloride channel, voltage-sensitive Ka | Voltage-sensitive chloride channel |
| *CLCNKB* | chloride channel, voltage-sensitive Kb | Voltage-sensitive chloride channel |
| *CLIC1* | chloride intracellular channel 1 | Chloride intracellular channel |
| *CLIC2* | chloride intracellular channel 2 | Chloride intracellular channel |
| *CLIC3* | chloride intracellular channel 3 | Chloride intracellular channel |
| *CLIC4* | chloride intracellular channel 4 | Chloride intracellular channel |
| *CLIC5* | chloride intracellular channel 5 | Chloride intracellular channel |
| *CLIC6* | chloride intracellular channel 6 | Chloride intracellular channel |
| *CNGA1* | cyclic nucleotide gated channel alpha 1 | Cyclic nucleotide-regulated channels |
| *CNGA2* | cyclic nucleotide gated channel alpha 2 | Cyclic nucleotide-regulated channels |
| *CNGA3* | cyclic nucleotide gated channel alpha 3 | Cyclic nucleotide-regulated channels |
| *CNGA4* | cyclic nucleotide gated channel alpha 4 | Cyclic nucleotide-regulated channels |
| *CNGB1* | cyclic nucleotide gated channel beta 1 C | yclic nucleotide-regulated channels |
| *CNGB3* | cyclic nucleotide gated channel beta 3 C | yclic nucleotide-regulated channels |
| *GABARAP* | GABA(A) receptor-associated protein | GABAA receptors |
| *GABRA1* | gamma-aminobutyric acid (GABA) A receptor, alpha 1 | GABAA receptors |
| *GABRA2* | gamma-aminobutyric acid (GABA) A receptor, alpha 2 | GABAA receptors |
| *GABRA3* | gamma-aminobutyric acid (GABA) A receptor, alpha 3 | GABAA receptors |
| *GABRA4* | gamma-aminobutyric acid (GABA) A receptor, alpha 4 | GABAA receptors |
| *GABRA5* | gamma-aminobutyric acid (GABA) A receptor, alpha 5 | GABAA receptors |
| *GABRA6* | gamma-aminobutyric acid (GABA) A receptor, alpha 6 | GABAA receptors |
| *GABRB1* | gamma-aminobutyric acid (GABA) A receptor, beta 1 G | ABAA receptors |
| *GABRB2* | gamma-aminobutyric acid (GABA) A receptor, beta 2 G | ABAA receptors |
| *GABRB3* | gamma-aminobutyric acid (GABA) A receptor, beta 3 G | ABAA receptors |
| *GABRD* | gamma-aminobutyric acid (GABA) A receptor, delta GA | BAA receptors |
| *GABRE* | gamma-aminobutyric acid (GABA) A receptor, epsilon | GABAA receptors |
| *GABRG1* | gamma-aminobutyric acid (GABA) A receptor, gamma 1 | GABAA receptors |
| *GABRG2* | gamma-aminobutyric acid (GABA) A receptor, gamma 2 | GABAA receptors |
| *GABRG3* | gamma-aminobutyric acid (GABA) A receptor, gamma 3 | GABAA receptors |
| *GABRP* | gamma-aminobutyric acid (GABA) A receptor, pi | GABAA receptors |
| *GABRQ* | gamma-aminobutyric acid (GABA) A receptor, theta | GABAA receptors |
| *GABRR1* | gamma-aminobutyric acid (GABA) A receptor, rho 1 | GABAA receptors |
| *GABRR2* | gamma-aminobutyric acid (GABA) A receptor, rho 2 | GABAA receptors |
| *GABRR3* | gamma-aminobutyric acid (GABA) A receptor, rho 3 | GABAA receptors |
| *GLRA1* | glycine receptor, alpha 1 | Glycine receptors |
| *GLRA2* | glycine receptor, alpha 2 | Glycine receptors |
| *GLRA3* | glycine receptor, alpha 3 | Glycine receptors |
| *GLRA4* | glycine receptor, alpha 4 | Glycine receptors |
| *GLRB* | glycine receptor, beta | Glycine receptors |
| *GRIA1* | glutamate receptor, ionotropic, AMPA 1 | Ionotropic glutamate receptors |
| *GRIA2* | glutamate receptor, ionotropic, AMPA 2 | Ionotropic glutamate receptors |
| *GRIA3* | glutamate receptor, ionotropic, AMPA 3 | Ionotropic glutamate receptors |
| *GRIA4* | glutamate receptor, ionotropic, AMPA 4 | Ionotropic glutamate receptors |
| *GRID1* | glutamate receptor, ionotropic, delta 1 | Ionotropic glutamate receptors |
| *GRID2* | glutamate receptor, ionotropic, delta 2 | Ionotropic glutamate receptors |
| *GRIK1* | glutamate receptor, ionotropic, kainate 1 | Ionotropic glutamate receptors |
| *GRIK2* | glutamate receptor, ionotropic, kainate 2 | Ionotropic glutamate receptors |
| *GRIK3* | glutamate receptor, ionotropic, kainate 3 | Ionotropic glutamate receptors |
| *GRIK4* | glutamate receptor, ionotropic, kainate 4 | Ionotropic glutamate receptors |
| *GRIK5* | glutamate receptor, ionotropic, kainate 5 | Ionotropic glutamate receptors |
| *GRIN1* | glutamate receptor, ionotropic, N-methyl D-aspartate 1 | Ionotropic glutamate receptors |
| *GRIN2A* | glutamate receptor, ionotropic, N-methyl D-aspartate 2A | Ionotropic glutamate receptors |
| *GRIN2B* | glutamate receptor, ionotropic, N-methyl D-aspartate 2B | Ionotropic glutamate receptors |
| *GRIN2C* | glutamate receptor, ionotropic, N-methyl D-aspartate 2C | Ionotropic glutamate receptors |
| *GRIN2D* | glutamate receptor, ionotropic, N-methyl D-aspartate 2D | Ionotropic glutamate receptors |
| *GRIN3A* | glutamate receptor, ionotropic, N-methyl-D-aspartate 3A | Ionotropic glutamate receptors |
| *GRIN3B* | glutamate receptor, ionotropic, N-methyl-D-aspartate 3B | Ionotropic glutamate receptors |
| *HCN1* | hyperpolarization activated cyclic nucleotide-gated potassium channel 1 | Cyclic nucleotide-regulated channels |
| *HCN2* | hyperpolarization activated cyclic nucleotide-gated potassium channel 1 | Cyclic nucleotide-regulated channels |
| *HCN3* | hyperpolarization activated cyclic nucleotide-gated potassium channel 1 | Cyclic nucleotide-regulated channels |
| *HCN4* | hyperpolarization activated cyclic nucleotide-gated potassium channel 1 | Cyclic nucleotide-regulated channels |
| *HTR3A* | 5-hydroxytryptamine (serotonin) receptor 3A, | ionotropic 5-HT3 receptors |
| *HTR3B* | 5-hydroxytryptamine (serotonin) receptor 3B, | ionotropic 5-HT3 receptors |
| *HTR3C* | 5-hydroxytryptamine (serotonin) receptor 3C, | ionotropic 5-HT3 receptors |
| *HTR3D* | 5-hydroxytryptamine (serotonin) receptor 3D, | ionotropic 5-HT3 receptors |
| *HTR3E* | 5-hydroxytryptamine (serotonin) receptor 3E, | ionotropic 5-HT3 receptors |
| *HVCN1* | hydrogen voltage-gated channel 1 | Voltage-gated proton channel |
| *KCNA1* | potassium voltage-gated channel, shaker-related subfamily, member 1 (episodic ataxia with myokymia) | Voltage-gated potassium channels |
| *KCNA10* | potassium voltage-gated channel, shaker-related subfamily, member 10 (episodic ataxia with myokymia) | Voltage-gated potassium channels |
| *KCNA2* | potassium voltage-gated channel, shaker-related subfamily, member 2 (episodic ataxia with myokymia) | Voltage-gated potassium channels |
| *KCNA3* | potassium voltage-gated channel, shaker-related subfamily, member 3 (episodic ataxia with myokymia) | Voltage-gated potassium channels |
| *KCNA4* | potassium voltage-gated channel, shaker-related subfamily, member 4 (episodic ataxia with myokymia) | Voltage-gated potassium channels |
| *KCNA5* | potassium voltage-gated channel, shaker-related subfamily, member 5 (episodic ataxia with myokymia) | Voltage-gated potassium channels |
| *KCNA6* | potassium voltage-gated channel, shaker-related subfamily, member 6 (episodic ataxia with myokymia) | Voltage-gated potassium channels |
| *KCNA7* | potassium voltage-gated channel, shaker-related subfamily, member 7 (episodic ataxia with myokymia) | Voltage-gated potassium channels |
| *KCNAB1* | potassium voltage-gated channel, shaker-related subfamily, beta member 1 | Voltage-gated potassium channels |
| *KCNAB2* | potassium voltage-gated channel, shaker-related subfamily, beta member 2 | Voltage-gated potassium channels |
| *KCNAB3* | potassium voltage-gated channel, shaker-related subfamily, beta member 3 | Voltage-gated potassium channels |
| *KCNB1* | potassium voltage-gated channel, Shab-related subfamily, member 1 | Voltage-gated potassium channels |
| *KCNB2* | potassium voltage-gated channel, Shab-related subfamily, member 2 | Voltage-gated potassium channels |
| *KCNC1* | potassium voltage-gated channel, Shaw-related subfamily, member 1 | Voltage-gated potassium channels |
| *KCNC2* | potassium voltage-gated channel, Shaw-related subfamily, member 2 | Voltage-gated potassium channels |
| *KCNC3* | potassium voltage-gated channel, Shaw-related subfamily, member 3 | Voltage-gated potassium channels |
| *KCNC4* | potassium voltage-gated channel, Shaw-related subfamily, member 4 | Voltage-gated potassium channels |
| *KCND1* | potassium voltage-gated channel, Shal-related subfamily, member 1 | Voltage-gated potassium channels |
| *KCND2* | potassium voltage-gated channel, Shal-related subfamily, member 2 | Voltage-gated potassium channels |
| *KCND3* | potassium voltage-gated channel, Shal-related subfamily, member 3 | Voltage-gated potassium channels |
| *KCNE1* | potassium voltage-gated channel, Isk-related family, member 1 | Voltage-gated potassium channels |
| *KCNE1L* | KCNE1-like | Voltage-gated potassium channels |
| *KCNE2* | potassium voltage-gated channel, Isk-related family, member 2 | Voltage-gated potassium channels |
| *KCNE3* | potassium voltage-gated channel, Isk-related family, member 3 | Voltage-gated potassium channels |
| *KCNE4* | potassium voltage-gated channel, Isk-related family, member 4 | Voltage-gated potassium channels |
| *KCNF1* | potassium voltage-gated channel, subfamily F, member 1 | Voltage-gated potassium channels |
| *KCNG1* | potassium voltage-gated channel, subfamily G, member 1 | Voltage-gated potassium channels |
| *KCNG2* | potassium voltage-gated channel, subfamily G, member 2 | Voltage-gated potassium channels |
| *KCNG3* | potassium voltage-gated channel, subfamily G, member 3 | Voltage-gated potassium channels |
| *KCNG4* | potassium voltage-gated channel, subfamily G, member 4 | Voltage-gated potassium channels |
| *KCNH1* | potassium voltage-gated channel, subfamily H (eagrelated), member 1 | Voltage-gated potassium channels |
| *KCNH2* | potassium voltage-gated channel, subfamily H (eagrelated), member 2 | Voltage-gated potassium channels |
| *KCNH3* | potassium voltage-gated channel, subfamily H (eagrelated), member 3 | Voltage-gated potassium channels |
| *KCNH4* | potassium voltage-gated channel, subfamily H (eagrelated), member 4 | Voltage-gated potassium channels |
| *KCNH5* | potassium voltage-gated channel, subfamily H (eagrelated), member 5 | Voltage-gated potassium channels |
| *KCNH6* | potassium voltage-gated channel, subfamily H (eagrelated), member 6 | Voltage-gated potassium channels |
| *KCNH7* | potassium voltage-gated channel, subfamily H (eagrelated), member 7 | Voltage-gated potassium channels |
| *KCNH8* | potassium voltage-gated channel, subfamily H (eagrelated), member 8 | Voltage-gated potassium channels |
| *KCNJ1* | potassium inwardly-rectifying channel, subfamily J, member 1 | Inwardly rectifying potassium channels |
| *KCNJ10* | potassium inwardly-rectifying channel, subfamily J, member 10 | Inwardly rectifying potassium channels |
| *KCNJ11* | potassium inwardly-rectifying channel, subfamily J, member 11 | Inwardly rectifying potassium channels |
| *KCNJ12* | potassium inwardly-rectifying channel, subfamily J, member 12 | Inwardly rectifying potassium channels |
| *KCNJ13* | potassium inwardly-rectifying channel, subfamily J, member 13 | Inwardly rectifying potassium channels |
| *KCNJ14* | potassium inwardly-rectifying channel, subfamily J, member 14 | Inwardly rectifying potassium channels |
| *KCNJ15* | potassium inwardly-rectifying channel, subfamily J, member 15 | Inwardly rectifying potassium channels |
| *KCNJ16* | potassium inwardly-rectifying channel, subfamily J, member 16 | Inwardly rectifying potassium channels |
| *KCNJ18* | potassium inwardly-rectifying channel, subfamily J, member 18 | Inwardly rectifying potassium channels |
| *KCNJ2* | potassium inwardly-rectifying channel, subfamily J, member 2 | Inwardly rectifying potassium channels |
| *KCNJ3* | potassium inwardly-rectifying channel, subfamily J, member 3 | Inwardly rectifying potassium channels |
| *KCNJ4* | potassium inwardly-rectifying channel, subfamily J, member 4 | Inwardly rectifying potassium channels |
| *KCNJ5* | potassium inwardly-rectifying channel, subfamily J, member 5 | Inwardly rectifying potassium channels |
| *KCNJ6* | potassium inwardly-rectifying channel, subfamily J, member 6 | Inwardly rectifying potassium channels |
| *KCNJ8* | potassium inwardly-rectifying channel, subfamily J, member 8 | Inwardly rectifying potassium channels |
| *KCNJ9* | potassium inwardly-rectifying channel, subfamily J, member 9 | Inwardly rectifying potassium channels |
| *KCNK1* | potassium channel, subfamily K, member 1 | Two-P potassium channels |
| *KCNK10* | potassium channel, subfamily K, member 10 | Two-P potassium channels |
| *KCNK12* | potassium channel, subfamily K, member 12 | Two-P potassium channels |
| *KCNK13* | potassium channel, subfamily K, member 13 | Two-P potassium channels |
| *KCNK15* | potassium channel, subfamily K, member 15 | Two-P potassium channels |
| *KCNK16* | potassium channel, subfamily K, member 16 | Two-P potassium channels |
| *KCNK17* | potassium channel, subfamily K, member 17 | Two-P potassium channels |
| *KCNK18* | potassium channel, subfamily K, member 18 | Two-P potassium channels |
| *KCNK2* | potassium channel, subfamily K, member 2 | Two-P potassium channels |
| *KCNK3* | potassium channel, subfamily K, member 3 | Two-P potassium channels |
| *KCNK4* | potassium channel, subfamily K, member 4 | Two-P potassium channels |
| *KCNK5* | potassium channel, subfamily K, member 5 | Two-P potassium channels |
| *KCNK6* | potassium channel, subfamily K, member 6 | Two-P potassium channels |
| *KCNK7* | potassium channel, subfamily K, member 7 | Two-P potassium channels |
| *KCNK9* | potassium channel, subfamily K, member 9 | Two-P potassium channels |
| *KCNMA1* | potassium large conductance calcium-activated channel, subfamily M, alpha member 1 | Calcium-activated potassium channels |
| *KCNMB1* | potassium large conductance calcium-activated channel, subfamily M, beta member 1 | Calcium-activated potassium channels |
| *KCNMB2* | potassium large conductance calcium-activated channel, subfamily M, beta member 2 | Calcium-activated potassium channels |
| *KCNMB3* | potassium large conductance calcium-activated channel, subfamily M, beta member 3 | Calcium-activated potassium channels |
| *KCNMB4* | potassium large conductance calcium-activated channel, subfamily M, beta member 4 | Calcium-activated potassium channels |
| *KCNN1* | potassium intermediate/small conductance calciumactivated channel, subfamily N, member 1 | Calcium-activated potassium channels |
| *KCNN2* | potassium intermediate/small conductance calciumactivated channel, subfamily N, member 2 | Calcium-activated potassium channels |
| *KCNN3* | potassium intermediate/small conductance calciumactivated channel, subfamily N, member 3 | Calcium-activated potassium channels |
| *KCNN4* | potassium intermediate/small conductance calciumactivated channel, subfamily N, member 4 | Calcium-activated potassium channels |
| *KCNQ1* | potassium voltage-gated channel, KQT-like subfamily, member 1 | Voltage-gated potassium channels |
| *KCNQ2* | potassium voltage-gated channel, KQT-like subfamily, member 2 | Voltage-gated potassium channels |
| *KCNQ3* | potassium voltage-gated channel, KQT-like subfamily, member 3 | Voltage-gated potassium channels |
| *KCNQ4* | potassium voltage-gated channel, KQT-like subfamily, member 4 | Voltage-gated potassium channels |
| *KCNQ5* | potassium voltage-gated channel, KQT-like subfamily, member 5 | Voltage-gated potassium channels |
| *KCNS1* | potassium voltage-gated channel, delayed-rectifier, subfamily S, member 1 | Voltage-gated potassium channels |
| *KCNS2* | potassium voltage-gated channel, delayed-rectifier, subfamily S, member 2 | Voltage-gated potassium channels |
| *KCNS3* | potassium voltage-gated channel, delayed-rectifier, subfamily S, member 3 | Voltage-gated potassium channels |
| *KCNT1* | potassium channel, subfamily T, member 1 | Calcium-activated potassium channels |
| *KCNT2* | potassium channel, subfamily T, member 2 | Calcium-activated potassium channels |
| *KCNU1* | potassium channel, subfamily U, member 1 | Calcium-activated potassium channels |
| *KCNV1* | potassium channel, subfamily V, member 1 | Voltage-gated potassium channels |
| *KCNV2* | potassium channel, subfamily V, member 2 | Voltage-gated potassium channels |
| *MCOLN1* | mucolipin 1 | Transient receptor potential channels |
| *MCOLN2* | mucolipin 2 | Transient receptor potential channels |
| *MCOLN3* | mucolipin 3 | Transient receptor potential channels |
| *NALCN* | sodium leak channel, non-selective | Voltage-independent cation channels |
| *P2RX1* | purinergic receptor P2X, ligand-gated ion channel, 1 | P2X receptors |
| *P2RX2* | purinergic receptor P2X, ligand-gated ion channel, 2 | P2X receptors |
| *P2RX3* | purinergic receptor P2X, ligand-gated ion channel, 3 | P2X receptors |
| *P2RX4* | purinergic receptor P2X, ligand-gated ion channel, 4 | P2X receptors |
| *P2RX5* | purinergic receptor P2X, ligand-gated ion channel, 5 | P2X receptors |
| *P2RX6* | purinergic receptor P2X, ligand-gated ion channel, 6 | P2X receptors |
| *P2RX7* | purinergic receptor P2X, ligand-gated ion channel, 7 | P2X receptors |
| *PKD1* | polycystic kidney disease 1 (autosomal dominant) | Transient receptor potential channels |
| *PKD2* | polycystic kidney disease 2 (autosomal dominant) | Transient receptor potential channels |
| *PKD2L1* | polycystic kidney disease 2-like 1 | Transient receptor potential channels |
| *PKD2L2* | polycystic kidney disease 2-like 2 | Transient receptor potential channels |
| *SCN10A* | sodium channel, voltage-gated, type X, alpha subunit | Voltage-gated sodium channels |
| *SCN11A* | sodium channel, voltage-gated, type XI, alpha subunit | Voltage-gated sodium channels |
| *SCN1A* | sodium channel, voltage-gated, type I, alpha subunit | Voltage-gated sodium channels |
| *SCN1B* | sodium channel, voltage-gated, type I, beta subunit | Voltage-gated sodium channels |
| *SCN2A* | sodium channel, voltage-gated, type II, alpha subunit | Voltage-gated sodium channels |
| *SCN2B* | sodium channel, voltage-gated, type II, beta subunit | Voltage-gated sodium channels |
| *SCN3A* | sodium channel, voltage-gated, type III, alpha subunit | Voltage-gated sodium channels |
| *SCN3B* | sodium channel, voltage-gated, type III, beta subunit | Voltage-gated sodium channels |
| *SCN4A* | sodium channel, voltage-gated, type IV, alpha subunit | Voltage-gated sodium channels |
| *SCN4B* | sodium channel, voltage-gated, type IV, beta subunit | Voltage-gated sodium channels |
| *SCN5A* | sodium channel, voltage-gated, type V, alpha subunit | Voltage-gated sodium channels |
| *SCN7A* | sodium channel, voltage-gated, type VII, alpha subunit | Voltage-gated sodium channels |
| *SCN8A* | sodium channel, voltage gated, type VIII, alpha subunit | Voltage-gated sodium channels |
| *SCN9A* | sodium channel, voltage-gated, type IX, alpha subunit | Voltage-gated sodium channels |
| *SCNN1A* | sodium channel, non-voltage-gated 1 alpha subunit | Nonvoltage-gated sodium channels |
| *SCNN1B* | sodium channel, non-voltage-gated 1, beta subunit | Nonvoltage-gated sodium channels |
| *SCNN1D* | sodium channel, non-voltage-gated 1, delta subunit | Nonvoltage-gated sodium channels |
| *SCNN1G* | sodium channel, non-voltage-gated 1, gamma subunit | Nonvoltage-gated sodium channels |
| *TPCN1* | two pore segment channel 1 | CatSper and two-pore channels |
| *TPCN2* | two pore segment channel 2 | CatSper and two-pore channels |
| *TRPA1* | transient receptor potential cation channel, subfamily A, member 1 | Transient receptor potential channels |
| *TRPC1* | transient receptor potential cation channel, subfamily C, member 1 | Transient receptor potential channels |
| *TRPC3* | transient receptor potential cation channel, subfamily C, member 3 | Transient receptor potential channels |
| *TRPC4* | transient receptor potential cation channel, subfamily C, member 4 | Transient receptor potential channels |
| *TRPC5* | transient receptor potential cation channel, subfamily C, member 5 | Transient receptor potential channels |
| *TRPC6* | transient receptor potential cation channel, subfamily C, member 6 | Transient receptor potential channels |
| *TRPC7* | transient receptor potential cation channel, subfamily C, member 7 | Transient receptor potential channels |
| *TRPM1* | transient receptor potential cation channel, subfamily M, member 1 | Transient receptor potential channels |
| *TRPM2* | transient receptor potential cation channel, subfamily M, member 2 | Transient receptor potential channels |
| *TRPM3* | transient receptor potential cation channel, subfamily M, member 3 | Transient receptor potential channels |
| *TRPM4* | transient receptor potential cation channel, subfamily M, member 4 | Transient receptor potential channels |
| *TRPM5* | transient receptor potential cation channel, subfamily M, member 5 | Transient receptor potential channels |
| *TRPM6* | transient receptor potential cation channel, subfamily M, member 6 | Transient receptor potential channels |
| *TRPM7* | transient receptor potential cation channel, subfamily M, member 7 | Transient receptor potential channels |
| *TRPM8* | transient receptor potential cation channel, subfamily M, member 8 | Transient receptor potential channels |
| *TRPV1* | transient receptor potential cation channel, subfamily V, member 1 | Transient receptor potential channels |
| *TRPV2* | transient receptor potential cation channel, subfamily V, member 2 | Transient receptor potential channels |
| *TRPV3* | transient receptor potential cation channel, subfamily V, member 3 | Transient receptor potential channels |
| *TRPV4* | transient receptor potential cation channel, subfamily V, member 4 | Transient receptor potential channels |
| *TRPV5* | transient receptor potential cation channel, subfamily V, member 5 | Transient receptor potential channels |
| *TRPV6* | transient receptor potential cation channel, subfamily V, member 6 | Transient receptor potential channels |
| *VDAC1* | voltage-dependent anion channel 1 | voltage-dependent anion channel |
| *VDAC2* | voltage-dependent anion channel 2 | voltage-dependent anion channel |
| *VDAC3* | voltage-dependent anion channel 3 | voltage-dependent anion channel |
| *ZACN* | zinc activated ligand-gated ion channel | ZAC |
