# Supplementary material for: A three ion channel genes-based signature predicts prognosis of primary glioblastoma patients and reveals a chemotherapy sensitive subtype
Source: Oncotarget. 2016 Oct 4;7(46):74895–903. doi: 10.18632/oncotarget.12462 (PMC5342710; doi:10.18632/oncotarget.12462)
Supplement: Supplementary file 3 [file oncotarget-07-74895-s003.docx]

**Table S4. 424 genes with increased expression in high risk group.**

| PLAUR | ANGPTL4 | MRC2 | QSOX1 | CCDC102A | IFI16 |
| --- | --- | --- | --- | --- | --- |
| COL6A2 | HSPA6 | PDIA4 | LAPTM4A | EMP1 | FBLN7 |
| RELB | SLC10A3 | HEXA | MSN | NUCB1 | LZTS1 |
| IFNGR2 | LOX | HSD3B7 | SPON2 | KDELC2 | NRP2 |
| PFN1 | RPN1 | REXO2 | VIM | TRABD | SEC61A1 |
| BCL3 | ZDHHC18 | TMEM8A | OSMR | ZNF217 | TNFAIP3 |
| SOCS3 | GLIPR1 | SERTAD3 | WTIP | NFKBIZ | MTMR11 |
| PMM2 | ICAM1 | PDLIM1 | KDELR2 | MIF | GBP1 |
| MANF | LOXL1 | XBP1 | MYH9 | ATP13A3 | CD151 |
| CLIC1 | CALR | C1RL | KCNE4 | IQGAP1 | ADAM9 |
| SPAG4 | EMP3 | BGN | COL5A2 | GGCT | DPP4 |
| S100A11 | TPM2 | ANXA1 | ZNF394 | MAPKAPK2 | EHD4 |
| FAM20C | LOC541471 | BCL10 | ZDHHC5 | TPM4 | ITPRIPL2 |
| TIMP1 | LUM | TNC | EMILIN2 | CASP4 | EMR2 |
| TNFRSF12A | CA9 | IGFBP2 | FNDC3B | SLC30A7 | SRD5A1 |
| ISG20 | C15orf48 | FKBP10 | PDLIM7 | CHPF2 | SLC4A7 |
| VKORC1 | MICALL2 | NOD1 | PLOD2 | ERRFI1 | HLA-A |
| PLK3 | IL10RB | CNN2 | BAX | RPL28 | GPC4 |
| PLP2 | TCIRG1 | PLOD3 | IMPDH1 | PXDN | GUSB |
| PHLDA2 | SRPX2 | TRIP10 | CNIH3 | GNS | ERI1 |
| IFI30 | TYMP | FADD | IBSP | CTSA | ST8SIA4 |
| RCN3 | STBD1 | TNFRSF1B | GBE1 | CARS2 | CIB1 |
| TMSB10 | C19orf10 | CD248 | CSNK1G2 | PCSK5 | SOD2 |
| CFLAR | TMED9 | SOCS1 | CFI | RBMS1 | LATS2 |
| LGALS1 | TNFRSF1A | KDELR1 | C6orf141 | GALNS |  |
| ANXA2 | CLEC2B | SERPINA1 | ASPN | LTBP3 |  |
| FOSL1 | BRI3 | AIM1 | ADAMTSL4 | COL4A1 |  |
| TWF2 | TMEM158 | ITPKC | HSP90B1 | CHRNA9 |  |
| STC1 | COL1A2 | CLEC5A | UPP1 | CRTAP |  |
| TNFRSF14 | DNAJB1 | CDCP1 | LOC154761 | F11R |  |
| VAMP5 | MXRA5 | MMP14 | PLIN2 | C1orf85 |  |
| IFRD2 | ABCC3 | MMP9 | ARPC2 | MGAT4B |  |
| ICAM3 | COL3A1 | EFEMP2 | EFNB2 | MCL1 |  |
| SLC16A3 | RGS16 | BCKDK | IGFBP3 | DIRAS3 |  |
| SERPINE1 | COL5A1 | TNFRSF10B | FSTL1 | ARL4C |  |
| COL6A1 | IL8 | NAMPT | FAM129A | ESM1 |  |
| TRADD | ITGA3 | CTSC | TGFB1 | CD93 |  |
| P4HB | ETHE1 | SSR3 | HLA-B | PTPN9 |  |
| RRAS | BACE2 | ITGB3 | PABPC4 | LRRN4CL |  |
| PTX3 | PGK1 | CDR2 | PMEPA1 | SQRDL |  |
| THBD | SLC16A10 | ADAM12 | NR2F6 | SPOCD1 |  |
| RDH10 | OLFML2B | CXCL3 | COL4A2 | FKBP11 |  |
| COL1A1 | SLC20A1 | CD63 | PLAU | GBP2 |  |
| DCBLD2 | LAMB1 | ETV6 | TRIP6 | DSE |  |
| NNMT | NTAN1 | GALM | TRAM2 | PLEKHA4 |  |
| RAC2 | GPX8 | ACTA2 | SLC27A3 | FAM129B |  |
| LOXL2 | CCDC109B | CHST2 | DPYD | LMAN1 |  |
| MYL12A | PDIA5 | RARS | RAB27A | SP100 |  |
| GDF15 | WIPI1 | ADM | COL6A3 | FUCA2 |  |
| TGFBI | MAFF | FAM50A | FZD1 | ACTN4 |  |
| SERTAD1 | CHSY1 | SERPINH1 | MAP2K3 | ZYX |  |
| CEBPD | S100A10 | ARSJ | FZD7 | PRF1 |  |
| IER5L | CD44 | SMS | CTHRC1 | SLC22A18 |  |
| DDOST | CSTA | SP140L | PTPN2 | ARSD |  |
| EMILIN1 | CYR61 | FOSL2 | RCAN1 | RAB13 |  |
| ARID5A | CARD16 | PLXND1 | BATF3 | CD276 |  |
| CRIP1 | REEP4 | C1S | PPIC | ENG |  |
| LY96 | SAT1 | SYDE1 | LOXL3 | LGALS3 |  |
| G0S2 | GLT25D1 | TAGLN | C8orf4 | PLXNB2 |  |
| ARPC1B | RAP2B | C11orf24 | LAMC1 | EPHB4 |  |
| C1R | TEAD3 | NUDT19 | TNKS1BP1 | CMTM3 |  |
| UAP1 | CAPZA1 | SEC24D | ZFP36L2 | NEDD9 |  |
| MBD2 | PDIA3 | TOR1B | TNFAIP8 | WWTR1 |  |
| PRDX4 | THBS1 | EHD2 | S100A6 | MAP3K6 |  |
| LEPRE1 | TSPAN4 | SLC25A43 | FAM114A1 | VDR |  |
| IGFBP4 | SLC25A37 | LIF | TSPO | CHPF |  |
| S100A4 | JUN | GADD45A | SNAI2 | SLC25A19 |  |
| MYL9 | C1QTNF6 | SDF4 | RIPK1 | CA12 |  |
| HEXB | DUSP5 | ZNF600 | SIL1 | LAMB2 |  |
| HSPA5 | PVRL2 | SLC2A4RG | ALG3 | FAM46A |  |
| PTRF | TUBB6 | EPHA2 | SDC1 | ADPGK |  |
| PCOLCE | TMEM43 | ALG2 | NRP1 | PLS3 |  |
| EIF4EBP1 | GADD45B | ARF6 | MIR155HG | PLSCR1 |  |
| CKAP4 | PPIB | SHKBP1 | GALNT2 | B3GNT5 |  |
| DRAM1 | IRF1 | ERO1L | RAB32 | ITGA4 |  |
| KDELR3 | LMAN2 | GMPPB | ERP44 | TXNDC5 |  |
| MDK | RBPMS | RUNX1 | KLF10 | PTP4A3 |  |
| CHI3L1 | CSDA | SDF2L1 | PLA2G5 | FN1 |  |
| ITGA5 | ACTN1 | PML | TM9SF1 | SLC12A7 |  |
| SYNPO | PLOD1 | CXCR4 | HAS2 | MYO1C |  |
